# Supplementary material for: A Comprehensive Assessment of Ultraviolet-Radiation-Induced Mutations in Flammulina filiformis Using Whole-Genome Resequencing
Source: J Fungi (Basel). 2024 Mar 20;10(3):228. doi: 10.3390/jof10030228 (PMC10971301; doi:10.3390/jof10030228)
Supplement: Supplementary file 1 [file jof-10-00228-s001.zip › Supplementary Material S8/KEGG annotation/out/64381550635650.os/KO/out_map/map03440.html]

KEGG PATHWAY: Homologous recombination - Reference pathway


|  |  |
| --- | --- |
| **Homologous recombination - Reference pathway** |  |

[
Pathway menu
| Organism menu
| Pathway entry
| Show description
| User data mapping
]

Homologous recombination (HR) is essential for the accurate repair of DNA double-strand breaks (DSBs), potentially lethal lesions. HR takes place in the late S-G2 phase of the cell cycle and involves the generation of a single-stranded region of DNA, followed by strand invasion, formation of a Holliday junction, DNA synthesis using the intact strand as a template, branch migration and resolution. It is investigated that RecA/Rad51 family proteins play a central role. The breast cancer susceptibility protein Brca2 and the RecQ helicase BLM (Bloom syndrome mutated) are tumor suppressors that maintain genome integrity, at least in part, through HR.

|  |  |
| --- | --- |
| Reference pathway | 100% |
